# Diabetes in the News: Readability Analysis of Malaysian Diabetes Corpus

**DOI:** 10.3390/ijerph19116802

**Published:** 2022-06-02

**Authors:** Afendi Hamat, Azhar Jaludin, Tuti Ningseh Mohd-Dom, Haslina Rani, Nor Aini Jamil, Aznida Firzah Abdul Aziz

**Affiliations:** 1 Faculty of Social Sciences and Humanities, National University of Malaysia, Bangi 43600, Malaysia; fendi@ukm.edu.my (A.H.); azharj@ukm.edu.my (A.J.); 2 Faculty of Dentistry, National University of Malaysia, Kuala Lumpur 50300, Malaysia; hr@ukm.edu.my; 3 Faculty of Health Sciences, National University of Malaysia, Kuala Lumpur 50300, Malaysia; ainijamil@ukm.edu.my; 4 Faculty of Medicine, National University of Malaysia, Kuala Lumpur 50300, Malaysia; draznida@ppukm.ukm.edu.my

**Keywords:** readability, health literacy, health promotion, health communication, diabetes education, diabetes prevention

## Abstract

This paper describes a study to evaluate the readability scores of Malaysian newspaper articles meant to create awareness of diabetes among the public. In contrast to patient-specific sources of information, mass media may potentially reach healthy people, thus preventing them from becoming part of the diabetes statistics. Articles published within a selected corpus from the years 2013 to 2018 and related to awareness regarding diabetes were sampled, and their readability was scored using Flesch Kinkaid Reading Ease (FKRE). Features of three articles ranked as the best and worst for readability were qualitatively analyzed. The average readability for the materials is low at 49.6 FKRE, which may impede the uptake of information contained in the articles. Feature analysis of articles with the best and worst readability indicates that medical practitioners may not be the best spokesperson to reach the public. It also indicates that simple sentence structures could help improve readability. There is still much room for improvement in attaining good public health literacy through mass media communication. Public health and media practitioners should be vigilant of the language aspects of their writing when reaching out to the public.

## 1. Introduction

The latest National Health and Morbidity Survey (NHMS) conducted by the Ministry of Health Malaysia [1] reported that nearly one in five Malaysian adults, or 18.3%, has diabetes. Malaysians exhibit high-risk traits for diabetes partly because 50% of adults are overweight or obese, and 95% do not consume an adequate intake of vegetables and fruits. Uncontrolled diabetes can lead to various health complications causing a significant burden on individuals, caregivers, and health care systems. A total of USD 600 million is estimated to have been expended annually to manage this disease in Malaysia [2]. Further, the same NHMS report estimated that 35% of Malaysian adults have low health literacy, drawing attention to the question of whether public health promotion initiatives such as diabetes awareness messages are readable by the general population.

Readability is an important issue in the dissemination of public health materials. Higher readability levels of these materials often lead to better retention of knowledge and better overall health literacy. This, in turn, translates into overall better outcomes in the sphere of public health: better health-related awareness and knowledge, fewer hospitalizations, and better adherence to medication and health-related recommendations. While readability could refer to various definitions, in this paper, it is defined as ‘the determination by systematic formulae of the reading comprehension level a person must have to understand written materials’ [3].

For chronic diseases such as diabetes mellitus, limited health literacy among patients and their families will lead to a higher burden of care [4]. Liu et al. (2020) define health literacy as the “ability of an individual to obtain and translate knowledge and information to maintain and improve health in a way that is appropriate to the individual and system contexts” [5]. It is within the first step of ‘obtain and translate knowledge and information’ that readability plays a critical role within the overall framework of health literacy. Although basic literacy (referring to the ability to read and write) is necessary for individuals to understand health information, it does not guarantee their ability to understand what is read, as it has been shown that low health literacy exists even in populations with high literacy levels [6]. Incidentally, despite the overall high literacy levels among the Malaysian population, their health literacy has been rated as poor [1,7].

Acknowledging the importance of population health literacy in efforts to curb chronic diseases such as diabetes, Malaysia has agreed to be a signatory to the Shanghai declaration endorsing health literacy as a critical determinant of health [8]. Nevertheless, Malaysia has yet to strengthen a wider adoption and commitment in efforts to improve health literacy among Malaysians. Conversely, health literacy research and practice are still in their early stages, with only a few Malaysian studies published on this subject [9]. Improvements in the health literacy of Malaysians can provide real hope for the country to halt the disturbingly rapid rise of diabetes among its population and subsequently reduce its burden on healthcare systems and communities.

### 1.1. Readability and Readability Formulas

Dale and Chall (1995) defined readability as “The total (including all the interactions) of all those elements within a given piece of printed material that affect the success a group of readers have [sic] with it [10]. The success is the extent to which they understand it, read it at an optimal speed, and find it interesting.” From the purely mathematical view of things, the last criteria proposed by Dale and Chall in 1995 may be impossible to calculate in any material [10]. Albright et al. in 1996 defined readability as “the determination by systematic formulae of the reading comprehension level a person must have to understand written materials” [3]. Later, McInnes and Haglund (2011) gave a simpler definition of readability as a “measure of the ease with which a passage of text can be read” [11]. The fact that there are varied definitions of the term is hardly surprising, as DuBay (2004) estimated that in the 1980s alone, there were about 200 readability formulas in use, each based on its own definition and principles regarding readability [12]. Regardless, readability formulas are the only available tools to quickly and economically assess the “ease with which a passage of text can be read”, according to McInnes and Haglund [11].

### 1.2. Previous Readability Studies on Health-Related Materials

Various studies have looked at the readability of materials related to diabetes and health literacy in general. Overland et al. (1993) studied 85 subjects with diabetes and how they read and comprehended different reading grades of health information. The researchers found that lowering the readability barriers improved patients’ ability to understand the materials [13]. Aguilera et al. (2010) studied the readability of 81 patient education materials on diabetes and concluded that the majority were not effective in reaching their audience because of the high readability barriers despite these materials being targeted at patients already living with diabetes [14].

At the turn of the century, more people turn to the internet as a source of information, and this includes health-related information [15]. Bernard et al. (2018) used specific search terms on three different search engines to simulate typical scenarios of patients looking for information on diabetes [16]. The researchers zoomed in on 42 websites and evaluated the readability of the information contained within these sites. They concluded that the readability, in general, was higher than the average American reading level. McInnes and Haglund (2011) carried out a similar study and came up with a similar conclusion: the readability of the information needed to be vastly improved [11]. Research of similar nature has been carried out in different languages as well, for example, Korean [17], Persian [18], and Spanish [19]. At the other end of the difficulty spectrum, Smith and co-workers (2017) evaluated the readability of diabetes-related medical journal articles for the layperson [20]. Not unexpectedly, they concluded that most of the articles would be beyond a layperson’s level of comprehension and suggested a suitable summary section to be included for the general public in medical journals. The tests used in the research mentioned are typical readability tests such as Flesch Kincaid Reading Ease (FKRE), Flesch Kincaid Grade Level (FKG), SMOG Index, and Gunning FOG. The tests are carried out using a combination of manual calculations and, more usually, online tools.

In summary, all the aforementioned research studies have two things in common. First, they concluded that readability is indeed a problem. Secondly, they refer to scenarios involving people living with diabetes. While targeting patients with diabetes is crucial, there are members of the public who are diabetes-free, and among these are those with a considerable risk of getting diabetes in the future. This portion of the population would rarely interact with materials specific to, and designed for, people with diabetes. The most logical avenue to reach them for the purpose of creating better awareness of the problem is through the mass media, where they expect to interact with ‘common, everyday’ materials. However, this potent vector of approach would be handicapped if the readability of the materials falls below what is suitable for public consumption.

In view of the value and reach of the mass media, this study aimed to evaluate if the readability of the sample data (newspaper articles) from the Malaysian Diabetes Corpus (MyDC) falls within the acceptable range for the public. The materials were chosen as they are publicly available newspaper articles. The layperson is deemed more likely to read these newspaper articles compared to diabetes-related information that is provided in educational pamphlets or websites. In short, these ‘mass-media’ materials usually have more reach than diabetes-related information put out by specific governmental bodies and NGOs. The study sought to answer the following questions: What is the average readability of the overall materials? What are the patterns for the readability of the materials in the years covered by MyDC? What are the features of the articles that ranked best and worst for readability?

## 2. Methodology

The methodology of this study involved three components: (1) selection of articles, (2) selection of best calculation method to measure article readability, and (3) analysis of the features of three articles ranked as the best and worst for readability.

### 2.1. Selection of Articles

A corpus is used to study aspects of language use and its relationship to various spheres of human activities. In linguistics, the term corpus is used to refer to a principled and systematic collection of electronic texts to represent ‘language in use’. The corpus used in this study is the Malaysian Diabetes Corpus (MyDC) which is meant to monitor the linguistic trends surrounding the word ‘diabetes’. The MyDC is a diachronic and dynamic corpus built up to monitor the trends around the word ‘diabetes’ in Malaysian newspapers. It currently consists of data from a single newspaper, which is in the English language but is expected to grow with available resources.

Articles are selected based on the search term “diabet*” and added to the corpus manually. For this investigation, the articles from MyDC are further filtered to meet two criteria based on the direction of the investigation: (i) the theme for the article is ‘Awareness’. This is established by a separate linguistic study of the same data, and (ii) the article is meant for the healthy public and not for people already living with diabetes. Articles excluded from the selection were sports or similar events organized by NGOs or governmental bodies, articles specifically for people with diabetes, articles regarding local issues such as the passing away of notables and product releases and promotions related to diabetes. The result of this selection process was 74 articles out of 212 in the corpus.

While the official language in Malaysia is Malay, the only available corpus on diabetes news coverage is in English. Future developments may include the development of diabetes corpora in Malay or other languages. However, in spite of having English as her second language, Malaysia has been rated as ‘high’ in non-native command of English, being ranked 26th globally for English language proficiency among non-native countries [21]. Furthermore, English is officially the second language and is taught as a compulsory subject at primary and secondary school levels for a total of 11 years of schooling. At tertiary levels, almost all Malaysian universities offer English for Specific Purposes (ESP) as compulsory or elective subjects.

### 2.2. Selection of Best Methods to Calculate the Articles’ Readability

The readability calculations were carried out using an online tool available at https://www.webfx.com/tools/read-able/ (accessed 10 May 2021) by feeding the articles one by one and recording the data manually. Automated readability scoring tools are deemed accurate compared to hand calculations [11]. The formulas available from the online tool are Flesch Kincaid Reading Ease (FKRE), Flesch Kincaid Grade Level (FKGL), Gunning Fog Score (GFS), SMOG Index, Coleman Liau Index (CLI), and Automated Reading Index (ARI).

The Flesch Kinkaid Grade Level and the Flesch Kincaid Reading Ease were initially developed for the US military for use in its training manuals and related publications in 1975 [22]. The FKRE evaluates readability in terms of percentile scores, where higher scores indicate better readability. The FKGL, on the other hand, represents readability in terms of US school system grade levels. The grading system is given in Table 1.

The Gunning Fog Score was developed in 1952 [10], updated several times, and also uses the grading system to indicate readability. The SMOG (Simple Measure of Gobbledygook) Index was developed in 1969 to simplify the process of calculating readability [23] and is also the favored tool for assessing the readability of healthcare materials [24]. The Coleman Liau Index (CLI) and the Automated Reading Index (ARI) differ from the other formulas described above by making use of character count in a word instead of detecting the syllables contained within the word.

In this study, the steps taken to process the data were: (1) calculate the correlation between FKRE and the syllabic-oriented formulas; and (2) calculate the correlation between FKRE and the character-focused formulas. The two steps described above would illustrate whether a strong correlation between the FKRE and the other formulas exists. Table 2 shows a strong negative correlation between the FKRE and the other formulas, regardless of whether the formulas are syllabic-oriented or character-oriented. The negative correlation can be explained by the fact that the easier the text (higher FKRE value), the lower the text’s grade (lower grade values). This helped to establish FKRE as a viable yardstick to use in readability measurement for the dataset.

### 2.3. Feature Analysis of the Three Best and Worst Articles for Readability

Two language experts described the features of the articles (3 best and 3 worst articles) independently based on active vs. passive constructions, the overall tone of the articles, and other surface features of the articles. Their descriptions were tagged and compared to each other. The initial agreement between the two sets of descriptions was evaluated using Cohen’s Kappa. The result was 0.87, indicating a strong level of agreement [25]. These descriptions were presented, cross-checked, and a consensus was reached between the two raters.

### 2.4. Data Analysis

Data entry and analysis were performed using Microsoft Excel to calculate the descriptive statistics and correlation analysis.

## 3. Results

At the time of writing, MyDC has 212 articles with a total of 134,024 tokens (words) and 10,904 word types (different words), which are presented in Table 3. The final sample comprised a total of 74 articles that met the selection criteria.

### 3.1. Readability of the Articles

The kurtosis and skewness values, in addition to the close values for mean and median, displayed in Table 4, suggest that the data are consistent with a normal distribution. Therefore, we can use the mean as the best measure of central tendency for the FKRE values of the dataset. The mean of 49.6 matches the category of ‘fairly difficult to read’ to ‘difficult to read’ based on the interpretation in Table 1, requiring Grade 12 and above to understand and not within the category of ‘plain English’ (60–70 range).

### 3.2. Patterns of the Readability of the Articles

**Figure 1:**
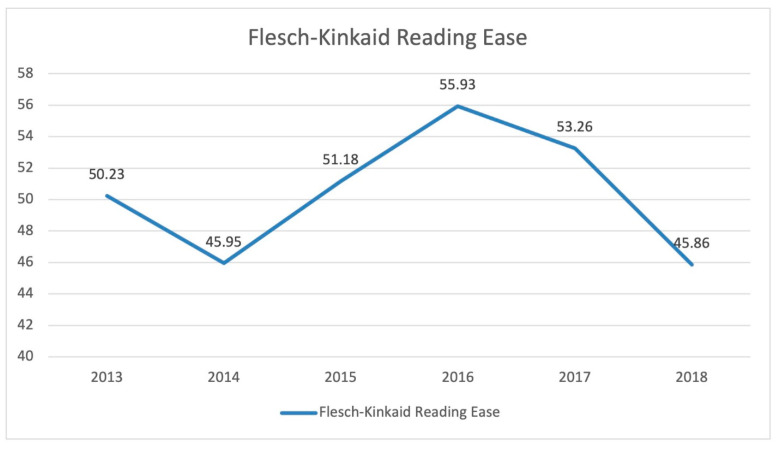
Trends of FKRE scores 2013–2018.

Figure 1 shows that, in general, there is not much variability in terms of FKRE values for the years measured. The range of 45–56 still puts the average readability of the materials in the “fairly difficult to read” to “difficult to read” category, as shown in Table 1.

### 3.3. Features of the Best and Worst Samples for Readability

The three best and three worst scoring articles (based on FKRE) were analyzed by the raters. The features of the six articles are illustrated in Table 5 for comparison. The best-ranked articles had been written in a conversational manner, with the use of simple active sentences as the most prevalent feature. Two out of three of the best articles involved laypersons talking about a healthy lifestyle and diabetes. In contrast, the worst-ranked articles contain features normally associated with low readability and comprehensibility, namely the use of dense passive sentences, numeric values, jargons, and lengthy sentences, as well as chemical names and numbers. Additionally, two out of three of the worst articles involved professional health workers talking about diabetes.

## 4. Discussion

In this study, we included selected samples of newspaper articles extracted from the Malaysian Diabetes Corpus and used an online tool to score and display their readability using Flesch Kinkaid Reading Ease (FKRE) as the main indicator. In doing so, we found that the average readability score of the sampled articles calculated in this study was 49.6, which falls under the “difficult to read” category. We compared this value with the readability values of Reader’s Digest and New York Times, which had been measured to be 65 (easily understood) and 52 (fairly difficult), respectively, because these two publications represent reading materials that are commonly accessed by the general population and cover a spectrum of various topics including health. Clearly, there is a general indication that the diabetes-related newspaper articles within the corpus may be too difficult for the average reader, as reflected by their lower score. Unfortunately, there are no readability scores of Malaysian public reading materials available to enable local comparisons.

Research has also pointed out that newspaper articles have different readability values based on content types. Flaounas et al. (2013) described a massive-scale readability analysis of English newspaper articles from 99 countries and ranked ‘Sports’ and ‘Arts’ at the top with a score of 54 and 48, respectively [26]. They listed 16 content types; however, ‘Health’ is not among them. Nevertheless, the average score for this dataset was 42, suggesting that the articles in our present study have higher readability compared to theirs, albeit it is still less than desirable if the objective is to create awareness about diabetes among the public.

With regards to the patterns of the articles’ readability seen over the years as captured in this study, the range of 45–56 is considered consistent, suggesting that the articles typically scored in the difficult to read category. The variance itself is not significant enough to provide any interesting discussion because of the low general readability of the materials. Furthermore, there were no recorded phenomena or events during the years covered to indicate any factors that may have been responsible for the slight variations. Then again, we captured slightly higher numbers than the average put forth by Flaounas et al. (2013), and at least for the year 2016, the average score has higher readability (55.9) than the best average (54 for Sports) reported in their study [26].

Our study recognizes that the best-ranked articles share similar textual features—written in a conversational manner and with heavy use of simple active sentences. Active sentences typically result in words with fewer syllables and are well-known to be simpler than their passive counterparts, making them more readable. The problems posed by the heavy use of passive sentence constructions to readability are well known and have been strongly criticized by John O’Hayre (1966) [27]. He came up with the Lensear Write Formula that factors in the use of active versus passive sentence constructions in evaluating readability. Although he meant it for government employees at the time, his words are still insightful for medical and public health practitioners today: “We know we can’t write simple, straightforward English without a lot of effort, so we automatically fall back on our technical jargon where we feel safest; this kind of writing is easiest for us to do” [27].

Also notable is the fact that two out of the three subjects are not medical or healthcare personnel. This may suggest that the layperson may be better articulated to convey information to the public. Medical experts may find it difficult at times to ‘speak plainly’ as their ‘normal’ language may already include terms or styles common to their professions (i.e., medical jargon). This is not a new problem. For example, in the field of e-learning, an instructional designer normally sits in between the subject matter expert (SME) and the learner. The instructional designer’s task is to redesign and reformat the knowledge from the SME into something easier and ‘consumable’ for the learner. Similarly, writers for the mass media as well as medical personnel communicating with the public via mass media should be aware that efforts must be made to simplify the language as much as possible. All healthcare practitioners will benefit from training in effective health communications as a part of their continuous professional development. Alternatively, such training may also be incorporated into the curriculum of healthcare professional programs.

When analyzing least readable articles, the article ‘Worst 1’ has an FKRE score of only 25, which is categorized as “very difficult to read”. This said article is a report of a scientific study, and thus, this finding is not surprising. However, the newspaper is meant to be read by the public, which means that low readability is not desirable. Smith, Buchanan, and McDonald’s (2017) suggestion that medical publications should include a layperson summary for each article could be of immense value in such cases [20]. Additionally, research reports that are of significance to the public would do well with a ‘press release’ version that can incorporate readability enhancements to make it easy for news outlets to publish.

There are limitations to this study. First, the Malaysian Diabetes Corpus is drawn from only one popular and established Malaysian English newspaper, and the potential exclusiveness of the selection may limit the representativeness of the study findings to writers of this particular newspaper and their targeted readership. Furthermore, there are many non-English newspapers available in Malaysia that may hypothetically differ in their readability levels yet remain to be explored due to the unavailability of the corpora in languages other than English. Secondly, the number of articles available for analysis is relatively small for a corpus. However, the small sample size is not considered a cause for major concern considering MyDC is a specialized corpus and is not meant to be used as a reference corpus like the British National Corpus, for example. Moreover, as a monitor corpus, its size and dimensions are expected to grow with time, depending on available resources. Thirdly, calculations for readability are mathematical approximates that evaluate the ‘readability’ of a piece of text quantitatively. As a strictly quantitative method, it yields usable information when covering large numbers of articles, but on the other hand, this method is very limited in its ability to drill down into the features of individual articles. To complement this limitation, the third research question was included to enrich our understanding of what makes a text more readable to the layperson in a more qualitative manner.

Based on the findings of this study, it is suggested that language meant for the public should be simplified as much as rationally possible. Efforts involving mass health communication were highly visible during the COVID-19 pandemic in Malaysia and elsewhere around the globe, and with generally good results. However, lifestyle diseases such as diabetes do not capture the public’s attention as much as COVID-19 did. Hence, it is even more important that the language used is simple enough for the public to understand and use to modify their behavior for a healthier lifestyle. This requires close coordination between news publishers on one hand and health authorities and experts on the other. Further research could establish guidelines for language readability to be used by news publishers with input and endorsement from the health authorities. Research is also needed for a more meaningful interpretation of readability scores. The FKRE readability formula used in this study relies on the American school system as a yardstick (FKGL). While its use eases the process of interpreting readability, a local and more relevant method of interpreting readability scores would be a tremendous boost to health communication. In fact, it can be of immense value to other forms of public communication where reading is a necessary part. This study used an English language corpus as its data; further research could expand the scope by including corpora of Malay and the vernacular languages used by the various ethnic groups in Malaysia. This effort would give a more detailed picture of a multi-lingual and diverse country such as Malaysia, whereby the identification of suitable linguistic approaches will help to convince the public to adopt and sustain healthy lifestyle modifications.

## 5. Conclusions

The evaluation of the sample data from MyDC shows that the readability of online newspaper articles meant to create awareness of diabetes among the public falls short of the acceptable ranges for easy reading. The average readability score of 49.6 FKRE, while ahead of the average in Flaounas et al. (2013) of 42, still leaves much room for improvement. It is possible that media practitioners are not aware of the potential of their medium to improve the diabetes situation, and hence, there is a need to work closely with public health practitioners. Flawed they may be, but readability formulas are currently the most readily available quantification tool available for public health experts and mass media practitioners to ensure materials on diabetes reach the public in a more effective manner.

## Figures and Tables

**Table 1:** Grading system and school levels (Flesch 1979).

Score (FKRE)	School Level (FKGL)	Notes
100.00–90.00	5th grade	Very easy to read. Easily understood by an average 11-year-old student.
90.0–80.0	6th grade	Easy to read. Conversational English for consumers.
80.0–70.0	7th grade	Fairly easy to read.
70.0–60.0	8th and 9th grade	Plain English. Easily understood by 13–15-year-old students.
60.0–50.0	From 10th to 12th grade	Fairly difficult to read.
50.0–30.0	College	Difficult to read.
30.0–10.0	College graduate	Very difficult to read. Best understood by university graduates.
10.0–0.0	Professional	Extremely difficult to read. Best understood by university graduates.

**Table 2:** Correlation between FKRE and the formulas.

	Flesch Kincaid Reading Ease
Flesch Kincaid Reading Ease	1
Flesch Kincaid Grade Level	−0.926910804
Gunning Fog Score	−0.83681993
SMOG Index	−0.917094037
Coleman Liau Index	−0.793461497
ARI	−0.845043078

**Table 3:** Number of articles included from year 2013 to 2018.

Year	Articles	Tokens (Words)	Token Types
2013	13	11,819	2300
2014	14	12,745	2767
2015	12	10,642	2191
2016	10	5514	1453
2017	8	5216	1492
2018	17	10,833	2350
Total	74	56,769	6122 (unique words)

**Table 4:** Descriptive statistics of the Flesh Kincaid Reading Ease (FKRE) Score.

Flesch Kincaid Reading Ease	
Mean	49.67
Standard error	1.04
Median	50.15
Mode	66.90
Standard deviation	8.92
Sample Variance	79.58
Kurtosis	−0.19
Skewness	0.01
Range	42.90
Minimum	25.60
Maximum	68.50
Sum	3675.70
Count	74
Confidence level (95.0%)	2.07

**Table 5:** Features of the three best and three worst scoring articles.

Best Articles	
Name	Features
Best 1	Reported and direct speech Subject: dieticians Text features: conversational, simple active sentences
Best 2	Letter to the editor Subject: none, citizen writer Text features: simple active sentences
Best 3	Reported and direct speech Subject: local actress Text features: conversational, simple active sentences
**Worst Articles**	
Name	Features
Worst 1	Research report Subject: reporting of DAWN2 research findings Text features: dense passive sentences, numeric values, jargons
Worst 2	Reported and direct speech Subject: NGOs and medical doctors Text features: conversational, explanatory, lengthy sentences, jargons
Worst 3	Reported and direct speech Subject: nutritionist Text features: passive sentences mixed with direct speech reporting, high density of chemical names and numbers

## Data Availability

The datasets used and analyzed during the current study are available from the corresponding author on reasonable request.
